# The complete mitochondrial genome of the fine flounder *Paralichthys adspersus* revealed by next-generation sequencing

**DOI:** 10.1080/23802359.2021.1914217

**Published:** 2021-08-31

**Authors:** Alan Marín, Edgar López-Landavery, Sophia González-Martinez, Lorenzo E. Reyes-Flores, Guillermo Corona-Herrera, Sandra Tapia-Morales, Carmen G. Yzásiga-Barrera, Juan I. Fernandino, Eliana Zelada-Mázmela

**Affiliations:** a Laboratorio de Genética, Fisiología y Reproducción, Facultad de Ciencias, Universidad Nacional del Santa, Chimbote, Peru; b Laboratorio de Biología del Desarrollo, Instituto Tecnológico de Chascomús, INTECH (CONICET-UNSAM), Chascomús, Argentina

**Keywords:** Mitogenome, Paralichthyidae, flatfish, NGS, Perú

## Abstract

The complete mitochondrial genome of the fine flounder *Paralichthys adspersus*, was determined for the first time through Next Generation Sequencing (NGS) approach. The mitogenome (GenBank accession no. MW288827) has 17,060 bp in length and consisted of the well-known 13 protein-coding genes, 22 tRNA genes, two rRNA genes, and the control region. The overall nucleotide composition of the whole mitogenome was A: 27.5%, C: 29.5%, G: 17.1%, and T: 25.9%. Phylogenetic analyses based on 12 protein-coding genes clustered *P. adspersus* in the monophyletic Paralichthyidae clade, showing the closest phylogenetic relationship with its congeneric species *P*. *olivaceus*.

The fine flounder, *Paralichthys adspersus* (Steindachner, 1867), is a commercially important species, which is distributed on the Pacific coast from Ecuador to Chile (Chirichigno and Cornejo 2001). In Peru, this species not only supports the most important artisanal flatfish fishery but also has great potential for aquaculture to local and international markets due to the high demand for its exquisite white flesh. Notwithstanding, the mitochondrial genome of *P. adspersus* remains unknown and to date, there is only one partial mitochondrial 16S rRNA gene sequence in the GenBank database (i.e. accession HM211198). Thus, the determination of the complete mitochondrial genome will be useful for the identification of potential DNA markers for aquaculture, biodiversity and population structure studies, and to obtain phylogenetic insights.

Specimens were collected from El Encanto beach (8°37'05.6"S 78°45'29.0"W, Virú, La Libertad, Peru, *n* = 3) and from an aquaculture facility Pacific Deep Frozen (Huarmey, Ancash, Peru, *n* = 4). Then, seven male specimens were transferred to the *Laboratorio of Genética, Fisiología y Reproducción* (*Universidad Nacional del Santa*, Ancash, Peru) and registered under the voucher numbers PA-CH1, PA-CH2, PA-CH3, PA-PDF1M, PA-PDF5M, PA-PDF6M, and PA-PDF12M. Each total genomic DNA was isolated from 10 mg of muscle tissue using an automated iPrep purification system (Invitrogen, Life Technologies; Thermo Fisher Scientific Inc., Waltham, MA, USA). DNA libraries were prepared using Nextera DNA Flex Library Prep (Illumina, San Diego, CA, USA) and paired-end (150 bp each) sequencing on a NextSeq 500 platform (Illumina, San Diego, CA, USA). Subsequently, the clean paired-end reads were assembled following the Norgal v1.0 tool pipeline (Al-Nakeeb et al. 2017), with minor modifications. For example, for the removal of sequencing adapters, Trimmomatic v0.36 (Bolger et al. 2014) was used instead of Adapter removal from Norgal. Trimming parameters were ILLUMINACLIP:illumina_all_adapters.fa:2:30:10 LEADING:5 TRAILING:5 SLIDINGWINDOW:5:25 MINLEN:50. Also, the maximum k-mer for megahit assembly was set to 100 instead of 105.

The complete circular mitogenome of *P. adspersus* presented 17,060 bp in size (GenBank accession no. MW288827), showing a nucleotide composition of: A 27.5%, C 29.5%, G 17.1%, and T 25.9%. Its mitogenome contains 13 protein-coding genes (PCGs) which were identified by an ORF finder analysis at NCBI (https://www.ncbi.nlm.nih.gov/orffinder/), and two ribosomal RNA genes were identified by sequence alignments and compared to several related species. Moreover, 22 transfer RNA genes were identified with tRNAscan-SE (Chan and Lowe 2019); and a control region (D-loop) of 1368 nt was also identified. All PCGs use the typical ATG start codon, except for the *COI* gene which utilizes the alternative GTG start codon. Six PCGs ended with the complete termination codons TAA (*ND1*, *ATPase 8*, *ND4L*, *ND5*, and *ND6*) or TAG (*COI*), whereas the remaining six PCGs used the incomplete stop codons TA– (*ND2*, *ATPase 6*, and *COIII*) or T–– (*COII*, *ND3*, *ND4*, and *Cytb*). These incomplete stop codons are presumably completed to TAA by posttranscriptional polyadenylation (Ojala et al. 1981). Except for *ND6* gene and eight tRNA genes (*tRNA-Gln*, *tRNA-Ala*, *tRNA-Asn*, *tRNA-Cys*, *tRNA-Tyr*, *tRNA-Ser*, *tRNA-Glu*, and *tRNA-Pro*), all other genes were located on the heavy strand (H-strand).

Finally, to infer the phylogenetic position of *P. adspersus*, a Bayesian analysis was performed based on the concatenated sequences of 12 PCGs (ND6 was excluded) from 12 pleuronectiform species using MrBayes 3.2 (Ronquist and Huelsenbeck 2003). Substitution saturation in single codon positions from each PCGs was analyzed using DAMBE5 (Xia 2013). Substitution model selection for all individual genes (12 PCGs) was performed using the software jModelTest 2 (Darriba et al. 2012). The best-fit substitution model for each partition was selected according to the Bayesian Information Criterion (BIC). As shown in the phylogenetic tree (Figure 1), six Paralichthyidae species included in the analysis constituted a monophyletic group, with *P. adspersus* gathered into a single subclade with *P. olivaceus*, while the clade formed by both species from the genus *Pseudorhombus* was placed at the most basal position of Paralichthyidae, reinforcing the data obtained from the present analysis.

**Figure 1.**
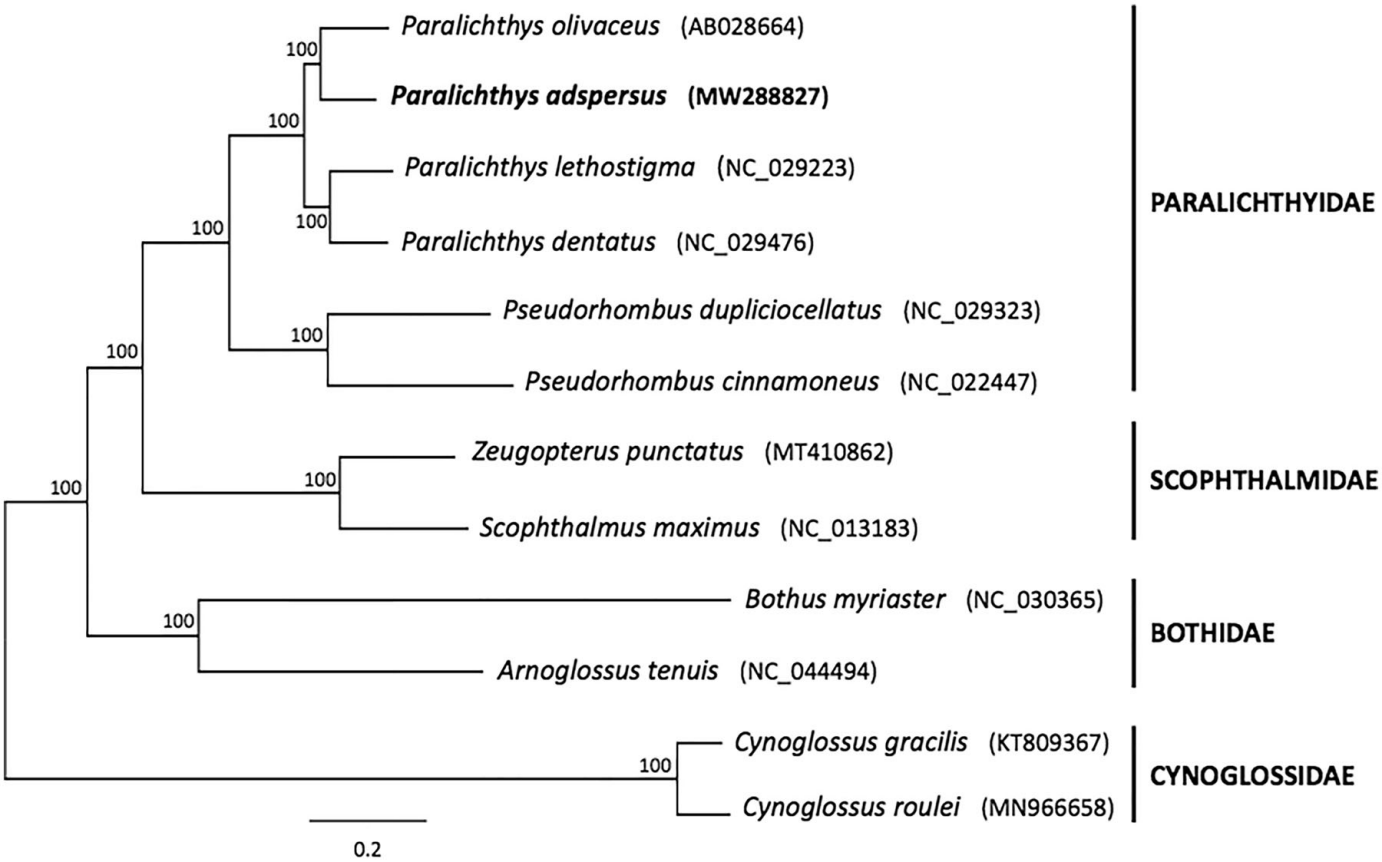
Bayesian phylogenetic tree inferred from 12 concatenated mitochondrial protein-coding genes (*ND6* was excluded) of pleuronectiform species. The sequence matrix (10,684 nt) used in the phylogenetic analyses consisted of unambiguously aligned regions of the first, second, and third codon positions (*COI* and *ATPase 8*), all other PCGs included only first and second codon positions. The GTR + I+G model was estimated as the best-fit substitution model for genes *ATPase6*, *COII*, *ND1*, *ND2*, and *ND5*; HKY + G model for genes *ATPase8* and COI; HKY + I+G model for genes *COIII*, *Cytb*, and *ND4*; and GTR + G model for genes *ND3* and *ND4L*. The position of *Paralichthys adspersus* (whose mitogenome was determined in this study) is shown in bold. Posterior probabilities at correspondent nodes are shown in percentages. GenBank accession numbers for each species are shown in parentheses.

## Data Availability

The data that support the findings of this study are openly available in [GenBank reference number MW288827, https://www.ncbi.nlm.nih.gov/nuccore/MW288827].
